# Adherence to physical activity guidelines in older adults, using objectively measured physical activity in a population-based study

**DOI:** 10.1186/1471-2458-14-382

**Published:** 2014-04-19

**Authors:** Barbara J Jefferis, Claudio Sartini, I-Min Lee, Minkyoung Choi, Antoinette Amuzu, Christina Gutierrez, Juan Pablo Casas, Sarah Ash, Lucy T Lennnon, S Goya Wannamethee, Peter H Whincup

**Affiliations:** 1 UCL Department of Primary Care & Population Health, UCL, London, UK; 2 UCL Physical Activity Research Group, London, UK; 3 Brigham and Women’s Hospital, Harvard Medical School, Boston, USA; 4 Department of Non-Communicable Disease Epidemiology, London School of Hygiene & Tropical Medicine, London, UK; 5 Institute of Cardiovascular Science, University College London, London, UK; 6 Division of Community Health Sciences, St George’s University of London, London, UK

**Keywords:** Older adults, Physical activity, Accelerometer, Physical health, Depression, Self-efficacy

## Abstract

**Background:**

Physical activity (PA) levels in older adults decline with age. The prevalence and correlates of adherence to current UK PA guidelines in older adults has not been studied using objectively measured PA, which can examine precisely whether PA is carried out in bouts of specified length and intensity.

**Methods:**

Free living men and women aged 70–93 years from 25 towns in the United Kingdom, participating in parallel on-going population based cohort studies were invited (by post) to wear a GT3x accelerometer over the hip for one week in 2010–12. Adherence to UK PA guidelines was defined as ≥150 minutes/week of moderate or vigorous PA (MVPA) in bouts of ≥10 minutes; the effect of different intensities and durations were examined.

**Results:**

1593 men and 857 women participated (responses 51% and 29% respectively). 15% men and 10% women achieved ≥150 minutes/week of MVPA (defined as >1040 cpm) in bouts lasting ≥10 minutes. With MVPA defined as >1952 cpm, prevalences were 7% and 3% respectively. Those adhering to guidelines were younger, had fewer chronic health conditions, less depression, less severe mobility limitations, but higher exercise self-efficacy and exercise outcomes expectations. They rated their local environment more highly for social activities and leisure facilities, having somewhere nice to go for a walk and feeling safe after dark, They left the house on more days per week, were more likely to use active transport (cycle or walk) and to walk a dog regularly.

**Conclusions:**

Few older adults attain current PA guidelines. Health promotion to extend the duration of moderate-intensity activity episodes to 10 minutes or more could yield important health gains among older adults. However future studies will need to clarify whether attaining guideline amounts of PA in spells lasting 10 minutes or more is critical for reducing chronic disease risks as well as improving cardiometabolic risk factors.

## Background

Low physical activity (PA) levels are associated with increased risks of many important chronic diseases and are an important global public health concern [1]. PA levels are particularly low in older people; in many countries (eg UK [2], other Europe [3] North Americas [4-6] and Brazil [7]), and levels decline steeply in older age. According to new UK guidelines, older adults should do at least 150 minutes per week of moderate-intensity activity or 75 minutes vigorous activity, or a combination of both, on most days of the week in bouts lasting 10 minutes or more [8]. These recommendations are consistent with other international guidelines [9]. Previous studies of attaining guidelines have raised concerns that adherence is low, but these have been mainly based on self-reported PA. Self-reported PA may be problematic among older adults, as their PA tends to be lighter in intensity and very variable in duration [10]. Such activity is hard to recall [11]. Very few large studies including the “oldest old” (over 80 years) use accelerometers to objectively measure PA. Accelerometers overcome problems of recall and reporting bias and, importantly, indicate whether moderate to vigorous intensity PA (MVPA) is accrued in bouts lasting 10 minutes or more, giving more accurate estimates of adherence to guidelines than self-reports.

We therefore aimed to estimate the prevalence of adherence to UK guidelines (which recommend carrying out 150 minutes of MVPA per week in bouts of at least 10 minutes), among community-dwelling older men and women. Secondly, we studied how altering the intensity of activity used to define MVPA (using 1040 and 1952 counts per minute [cpm]) and reducing bout-length (from 10 minutes to 5 minutes) impacted on attainment of 150 minutes of MVPA per week. We studied shorter bouts because the evidence for the total amount of PA required to reduce risk of death and disease is better than evidence for the need to do exercise in spells of particular duration [12]. We investigated different MVPA thresholds because there is limited data on this issue, particularly in the oldest old [13]. The third aim was to identify correlates of adherence to the guidelines. From a policy perspective, it is important to estimate the prevalence of adherence to the MVPA guidelines and what modifiable factors predict adherence. We reviewed previously published evidence about correlates of participation in PA (i.e., not just adherence to guidelines) in older adults to select a range of correlates from physical and mental health, exercise self-efficacy, trips outside the home, dog walking, and neighbourhood characteristics [14-17]. This study extends previous work as it is the largest UK-based study using accelerometers in 70–93 year olds to examine prevalence of adherence to guidelines alongside a wide range of correlates.

## Methods

### Sample

7735 men participating in the British Regional Heart Study, an on-going prospective, population-based cohort study were recruited from primary care centres in 24 British towns in 1978–80 and followed up repeatedly [18]. In 1999–2001, a parallel cohort study (British Women’s Heart Health Study) of 4286 women of the same age and in the same primary care centres was established, omitting two study towns (Dewsbury and Maidstone) and adding another (Bristol), with a response rate 60% [19]. In 2010–2012, 6529 survivors from these two cohorts (3292 men and 3237 women) were invited to participate in a study of objectively measured PA by post. Men were also asked to wear accelerometers on other occasions, here we use the postal survey that occurred concurrently with the women’s study and using the same study protocol. The National Research Ethics Service (NRES) Committee for London provided ethical approval. Participants provided informed written consent to the investigation, which was performed in accordance with the Declaration of Helsinki.

### Measures

#### ***Accelerometer data***

Participants were sent an accelerometer with a prepaid return envelope, a log diary and questionnaire (see below). Participants were asked to wear the GT3x accelerometer (Actigraph, Pensacola, Florida) over the right hip on an elasticated belt for 7 days, during waking hours, removing it for swimming or bathing. Data were processed using standard methods; raw data collected from movements registering on the vertical axis were integrated into 60 second increments periods (epochs). Non-wear time was identified and excluded using a commonly used and freely available R package “Physical Activity” [20]. Periods of continuous zeros lasting more than 90 minutes were assigned as non-wear time; short spells of non-zero counts lasting up to 2 minutes during the 90 minute period were allowed as non-wear time if no activity counts were detected during both the 30 minutes before and after that interval, to reflect the possibility of artefactual monitor movements (e.g. due to accidental movement of the monitor being disturbed while left on a table). This means that any non-zero counts except the allowed short interval of up to 2 minutes are considered as wear time. Valid wear days were defined as ≥600 minutes wear time, and participants with 3 or more valid days were included in analyses, a conventional requirement to estimate usual PA level [15,17,21]. High activity levels (>10,000 cpm) or high step counts (>20,000 steps/day) were verified against men’s daily log diaries. The number of minutes per day spent in PA of different intensity levels was categorised using count-based intensity threshold values of counts per minute developed for older adults [22]: <100 cpm for sedentary behavior (<1.5 MET),100-1040 for light activity (1.5-3 MET) and >1040 for MVPA,(≥3 MET). 1040 cpm is the favoured cut-point to define MVPA in this study as it was calibrated to identify moderate intensity activities (≥3 MET) in a sample of older adults [22], we also investigate the more widely used cut-point of 1952 cpm which was calibrated to identify moderate intensity activities (≥3 MET) in middle-aged adults [23].

#### ***Questionnaire data***

Participants completed a log diary detailing when they wore the monitor and a questionnaire including the following questions: self-rated health [excellent, good, fair or poor]; number of chronic health conditions (from a list of 12 conditions); falls history in the past 12 months; problems getting about outdoors [no difficulty, slight, moderate, severe or unable to do] (responses of moderate, severe or “unable to do” indicated mobility problems); the 4-point Geriatric Depression Scale (scores ≥2 indicated depression) [24]; the Lubben scale for social interaction (scores <12 indicated social isolation [25]). The Self-Efficacy for Exercise scale [26] and the Expected Outcomes for Habitual Exercise scale [26], which both ask 9 questions with responses across a likert scale, total scores were analysed as z-scores. Questions about facilities and safety of the local neighbourhood (with responses on a likert scale), included the social and leisure facilities for people like yourself; the facilities for people your age; the local transport for where you want to go; the area has somewhere nice to go for a walk; how safe from crime you feel when walking alone in the daytime and after dark. Participants reported whether most (food and household necessities) shopping was within easy walking distance (<15 minutes) of home, [yes, no, or someone else shops for me]; which forms of transport they used regularly [car, public transport, cycle, walk or other], cycle and walk were grouped together as “active transport”; how many days they left the house in the previous seven days [0–7] and if they regularly walked a dog [yes or no].

### Statistical methods

The total number of minutes spent in MVPA in each of the valid days (range 3–8 days) was summed. For participants who did not have 7 valid days, the total MVPA minutes were scaled to the number of valid days. Attaining ≥150 minutes of MVPA in 7 days was calculated for the total minutes of MVPA accumulated in bouts lasting (i) ≥5 minutes (MVPA5+) and (ii) ≥10 minutes (MVPA10+).

Descriptive statistics of demographic, social, and environmental variables stratified by gender were calculated and adjusted mean PA levels were estimated. Summary measures of demographic, social and environmental variables were calculated according to achieving ≥150 minutes MVPA5+ and MVPA10+. ANOVA and chi square tests were used to compare continuous and categorical variables, respectively.

Logistic regression models were used to compare participants who achieved guidelines to those who did not. Model 1 was adjusted for age, season, wear time (average minutes/day on valid days) and region of residence. Potential mediators were added one at a time to model 1, to evaluate the role of each. These included physical and mental health and wellbeing, perceptions of local environment and behavioural factors. Model 2 was additionally adjusted for mental and physical health (depression score and number of chronic conditions). Complete case analysis was used. As sensitivity analyses, the main analyses were repeated using a cut-point of >1952 cpm to define MVPA. Statistical analyses were run in R version 2.15.3 and Stata version 12.

## Results

**Figure 1:**
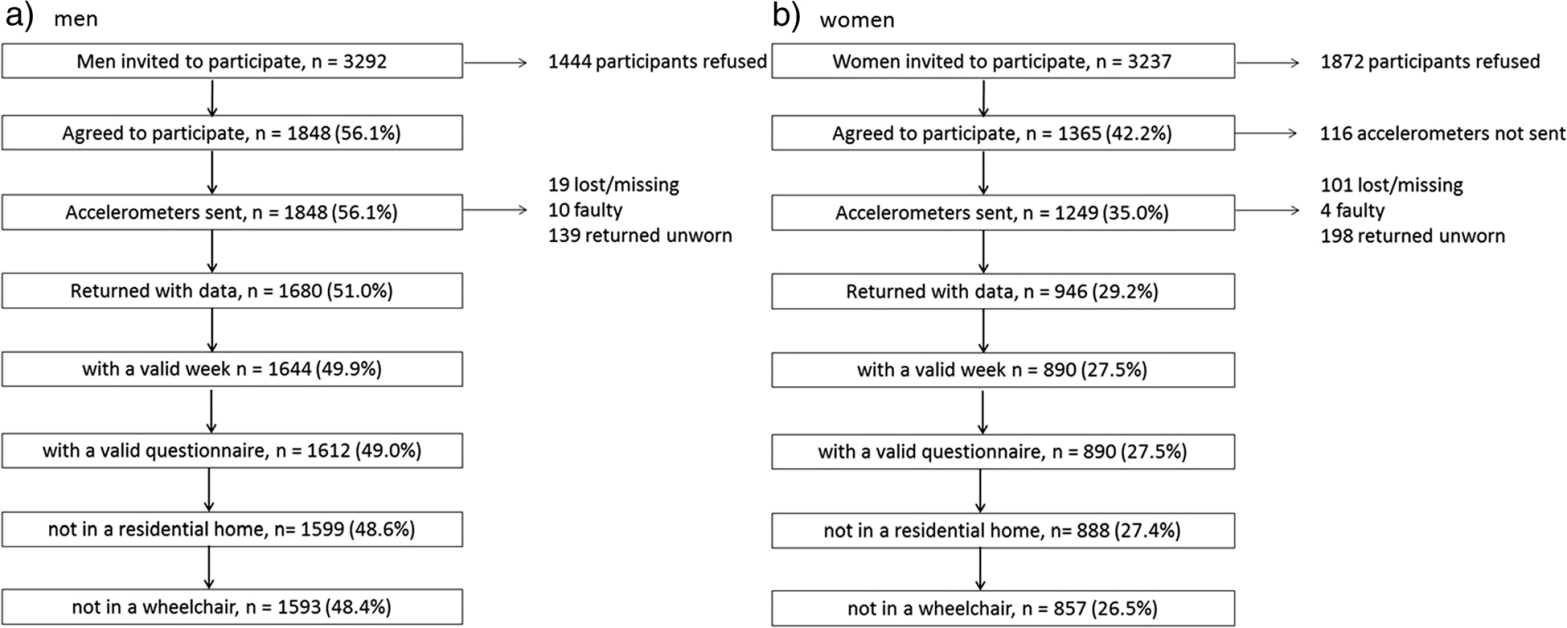
Recruitment flow chart and identification of the eligible population of men and women.

1680/3292 (51%) men and 946/3237 (29%) women agreed to participate and had accelerometer data, (Figure 1), of which 1644 men and 890 women had at least 3 days of 600 minutes wear per day and 32 men did not complete a questionnaire. Those in a residential home or confined to a wheelchair were excluded, leaving 1593 men and 857 women (total 2450) for analyses. Men and women who accepted the monitor compared to those who did not accept it had higher self-reported PA and lower BMI levels 10 years earlier and more often had manual rather than non-manual occupations. Table 1 presents the characteristics of the sample with valid data; participants were aged on average 78 (SD 4.7) years, range 69.9-93.3 years. Compared to women, men registered more accelerometer counts per day, more counts per minute, took more steps per day than women, spent more time in sedentary behaviour, less time in light activity and more time in MVPA. Among men, 17% (n = 269) did no bouts of MVPA lasting 5 minutes or more and 38% (n = 598) did no bouts of MVPA lasting 10 minutes or more during their week of monitoring; corresponding figures for women were 20% (n = 169) and 48% (n = 414).

**Table 1:** Characteristics of study participants, (n = 2540)

	**Men**	**Women**	**Total**	**p-value**^**1**^
N, % (n)	65.0(1593)	35.0(857)	100(2450)	
Age (years)				<0.001
70-75	28.2(450)	38.4(329)	31.8(779)	
>75-80	38.9(619)	30.1(258)	35.8(877)	
>80-85	23.1(368)	21.9(188)	22.7(556)	
Over 85	9.8(156)	9.6(82)	9.7(238)	
Living alone vs living with others, % (n)	21.4(338)	41.4(351)	28.4(689)	<0.001
**Physical health**				
≥3 Chronic conditions, % (n)	12.3(196)	10.7(92)	11.8(288)	<0.001
Falls in the past 12 months, % (n)				0.156
One fall	9.1(143)	10.8(91)	9.7(234)	
≥2 Falls	11.8(185)	9.7(82)	11.1(267)	
Moderate/severe mobility limitations outdoors, % (n)	14.1(220)	14.5(122)	14.2(342)	0.799
**Mental health and wellbeing**				
Exercise self efficacy, (z-scores), (mean, SD)	0.01(0.99)	0.03(0.99)	0.01(0.99)	0.622
Exercise outcome expectations, (z-scores), (mean, SD)	0(1.0)	0.03(0.95)	0.01(0.98)	0.380
Social isolation, (isolated), % (n)^2^	22.6(358)	20.4(174)	21.9(532)	0.206
Geriatric depression scale, (depressed), % (n)^3^	22.9(362)	20.8(177)	22.1(539)	0.232
**Environment**				
Very good/good social and leisure activities, % (n)^4^	50.6(781)	57.8(486)	53.1(1267)	<0.001
Very good/good facilities for people your age, % (n)^4^	43(658)	62.8(531)	50(1189)	<0.001
Very good/good Local transport, % (n)^4^	69.3(1066)	73.8(624)	70.9(1690)	0.020
The area has nice place to go for a walk, % (n)^4^	82.8(1301)	79.8(674)	81.7(1975)	0.064
Feel safe walking alone in daytime, % (n)^4^	96.4(1517)	95.7(814)	96.2(2331)	0.335
Feel safe walking alone after dark, % (n)^4^	70.3(1101)	35.8(305)	58.2(1406)	<0.001
**Behaviours**				
Leaves the house 5/days/week, %, (n)	84.3(1303)	73(609)	80.3(1912)	<0.001
Cycle/walk regularly, % (n)	55.3(881)	12.4(106)	40.3(987)	<0.001
Do most shopping walking distance from home, % (n)	33.2(515)	33.7(286)	33.4(801)	<0.001
Regularly walk a dog?, % (n)	11.3(177)	9.5(81)	10.6(258)	0.185
**PA levels/day, mean (95% CI)**^5^				
Counts/min	183(178, 188)	178(173, 183)	181(177, 185)	
Steps	4762(4638, 4887)	4470(4331, 4609)	4662(4567, 4757)	
Sedentary (minutes/day), <100 counts/min	625(621, 628)	582(577, 586)	609(607, 613)	
LIGHT (minutes/day), 100–1040 counts/min	195(192, 198)	221(217, 224)	203(201, 206)	
MVPA (minutes/day), >1040 counts/min	38(37, 40)	33(31, 34)	36(35, 37)	
**≥150 minutes/wk MVPA (MVPA >1040 cpm)**				
**≥**150 minutes/week of MVPA in bouts of 1+	62.3(992)	56.0(480)	60.1(1472)	0.003
**≥** 150 minutes/week of MVPA in bouts of 5+	28.4(452)	20.7(177)	25.7(629)	<0.001
**≥** 150 minutes/week of MVPA in bouts of 10+	15.4(245)	10.0(86)	13.5(331)	<0.001
**≥150 minutes/wk MVPA (MVPA >1952 cpm)**				
**≥** 150 minutes/week of MVPA in bouts of 1+	27.4(437)	18.9(162)	24.4(599)	<0.001
**≥** 150 minutes/week of MVPA in bouts of 5+	12.2(195)	7.6(65)	10.6(260)	<0.001
**≥** 150 minutes/week of MVPA in bouts of 10+	7.3(117)	3.0(26)	5.8(143)	<0.001

^1^Total counts comparisons of continuous and categorical variables were completed using ANOVA and chi square tests, respectively. Fisher exact tests were used for comparisons of categorical variables with less than five expected observations in any of the cells.

^2^Lubben scale, isolated <12.

^3^Geriatric Depression Scale, depressed >2.

^4^Self-rating of local area.

^5^Adjusted for average monitor wear time, day order, season, age and region. For total population (women + men) adjusted for average monitor wear time, day order, season, age and region and sex.

### Prevalence and correlates of attaining PA recommendations in bouts of 10 minutes or more

15% men (n = 245) and 10% women (n = 86) accumulated at least 150 minutes per week of MVPA in bouts of ≥10 minutes (MVPA10+) with MVPA defined as >1040 cpm (Table 2). Men and women who achieved MVPA10+ were more likely to be younger, have fewer chronic health conditions or severe mobility limitations, no falls in the past year(for men), higher exercise self-efficacy and exercise outcomes expectations and were less likely to be depressed. They rated their local environment more highly for social activities and leisure facilities, having somewhere nice to go for a walk and feeling safe after dark, and left the house on more days per week, and were more likely to regularly cycle or walk and to regularly walk a dog. Bivariate associations were confirmed in logistic regression models; Table 3, Model 1 (adjusting for age, region of residence, wear time, day order and season of wear), with the exception of falls history and local area social and leisure activities and facilities. In Model 2, adjusted additionally for depression and number of chronic conditions, meeting guidelines was most strongly associated with having no chronic health conditions compared to one or more, not being depressed or having mobility limitations, having higher exercise self-efficacy, feeling safe walking after dark, leaving the house more often, using active transport (walking or cycling) and regularly walking a dog.

**Table 2:** Characteristics of participants according attainment of 150 minutes/week MVPA (>1040 cpm) in bouts lasting 10 minutes or more

	**<150 mins/week MVPA**	**> = 150 mins/week MVPA**	**p-value**	**<150 mins/week MVPA**	**> = 150 mins/week MVPA**	**p-value**
N, % (n)	84.6(1348)	15.4(245)		90.0(771)	10.0(86)	
Age (years), % (n)			<0.001			<0.001
70-75	26(351)	40.4(99)		35.8(276)	61.6(53)	
>75-80	38.3(516)	42(103)		30.9(238)	23.3(20)	
>80-85	24.7(333)	14.3(35)		23(177)	12.8(11)	
>85	11(148)	3.3(8)		10.4(80)	2.3(2)	
Living alone vs living with others, % (n)	22(293)	18.6(45)	0.240	41.1(313)	44.2(38)	0.586
**Physical health**						
≥ 3 chronic conditions, % (n)	13.8(186)	4.1(10)	<0.001	11.5(89)	3.5(3)	0.003
Falls in the past 12 months, % (n)			<0.001			0.712
One fall	8.7(115)	11.5(28)		11.1(84)	8.2(7)	
≥ 2 falls	13.1(173)	4.9(12)		9.6(73)	10.6(9)	
Moderate/severe mobility limitations outdoors, % (n)	16.2(214)	2.5(6)	<0.001	15.9(121)	1.2(1)	<0.001
**Mental health and wellbeing**						
Exercise self efficacy, (z-scores), (mean, SD)	-0.12(1.00)	0.68(0.67)	<0.001	-0.07(0.97)	0.84(0.76)	<0.001
Exercise outcome expectations, (z-scores), (mean, SD)	-0.09(1.00)	0.50(0.87)	<0.001	-0.04(0.95)	0.66(0.71)	<0.001
Socially isolated, % (n)	23.4(313)	18.4(45)	0.081	20.7(159)	17.6(15)	0.504
Depressed (Geriatric depression scale), % (n)	25.6(343)	7.8(19)	<0.001	22.5(172)	5.8(5)	<0.001
**Environment**						
Social and leisure activities, % (n)^1^	48.8(636)	60.2(145)	0.001	56.6(427)	68.6(59)	0.032
Facilities for people your age, % (n)^1^	41.8(538)	49.2(120)	0.034	61.4(467)	74.4(64)	0.018
Local transport to where you want to go, % (n)^1^	69.1(898)	70.3(168)	0.720	74(563)	72.6(61)	0.787
The area has a nice place to go for a walk, % (n)^1^	81.4(1079)	90.6(222)	<0.001	78.7(597)	89.5(77)	0.017
Feel safe walking alone in the daytime, % (n)^1^	96.1(1276)	98.4(241)	0.076	95.3(729)	98.8(85)	0.165
Feel safe walking alone after dark, % (n)^1^	67.5(892)	85.7(209)	<0.001	32.9(252)	61.6(53)	<0.001
**Behaviours**						
Leave the house ≥5 days/past week, %, (n)	81.9(1068)	97.1(235)	<0.001	70.5(530)	96.3(79)	<0.001
Cycle/walk regularly, % (n)	51.4(693)	76.7(188)	<0.001	9.7(75)	36(31)	<0.001
Do most shopping walking distance from home, % (n)	32.6(427)	36.5(88)	0.116	32.3(247)	45.9(39)	0.034
Regularly walks a dog, % (n)	8.1(108)	28.6(69)	<0.001	6.4(49)	37.2(32)	<0.001

^1^Self-rating of local area as ’good” or “very good”.

**Table 3:** Associations (odds ratio, [95% CI]) between participant characteristics and attaining ≥150 minutes/week of MVPA(>1040 cpm) in bouts lasting 10 minutes or more, (n = 2426)

	**Men**		**Women**	
	**Model 1**^**5**^	**Model 2**^**6**^	**Model 1**^**5**^	**Model 2**^**6**^
**Physical health**				
No chronic conditions vs ≥1 condition (baseline)	1.97(1.48,2.61)	1.86(1.40,2.48)	1.63(1.02,2.62)	1.51(0.93,2.43)
One fall vs no falls in past 12 months	1.53(0.97,2.42)	1.69(1.06,2.69)	0.84(0.36,1.96)	0.95(0.41,2.22)
≥2 falls vs no falls in past 12 months	0.44(0.24,0.81)	0.59(0.31,1.11)	1.44(0.66,3.15)	2.00(0.90,4.45)
No/slight mobility limitations vs moderate or more	6.53(2.84,14.98)	4.00(1.70,9.38)	12.44(1.66,93.32)	8.08(1.07,60.93)
**Mental health and wellbeing**				
Exercise self efficacy, (z-scores, 1-SD increase)	2.66(2.19,3.23)	2.47(2.02,3.01)	2.52(1.89,3.37)	2.42(1.79,3.26)
Exercise outcome expectations, (z-scores, 1-SD increase)	1.85(1.58,2.17)	1.73(1.47,2.04)	2.42(1.76,3.34)	2.28(1.64,3.16)
Not socially isolated vs isolated^1^	1.17(0.82,1.67)	1.06(0.73,1.53)	0.83(0.45,1.54)	0.59(0.31,1.13)
Not depressed vs depressed ^2^	3.40(2.08,5.57)	3.21(1.96,5.27)	3.89(1.52,9.97)	3.66(1.43,9.40)
**Environment**				
Social and leisure activities^3^	1.48(1.11,1.97)	1.30(0.97,1.75)	1.54(0.94,2.52)	1.40(0.85,2.31)
Facilities for people your age^3^	1.25(0.94,1.66)	1.10(0.82,1.47)	1.70(1.01,2.87)	1.55(0.91,2.63)
Local transport^3^	1.04(0.76,1.42)	0.98(0.72,1.35)	0.83(0.49,1.41)	0.76(0.44,1.30)
The area has somewhere nice to go for a walk^3^	1.90(1.20,3.02)	1.65(1.03,2.63)	1.91(0.92,3.98)	1.77(0.84,3.71)
Feel safe when walking alone in the daytime^4^	2.15(0.76,6.10)	1.59(0.55,4.63)	2.50(0.33,19.07)	1.82(0.23,14.24)
Feel safe when walking alone after dark^4^	2.59(1.77,3.81)	2.31(1.56,3.41)	2.81(1.73,4.54)	2.51(1.54,4.09)
**Behaviours**				
Leave house ≥5 days/week vs < 5 days/week	6.03(2.78,13.08)	4.74(2.17,10.38)	7.38(2.26,24.09)	5.81(1.77,19.11)
Cycle/walk vs use car/public transport	2.71(1.95,3.75)	2.32(1.67,3.24)	4.92(2.89,8.36)	4.45(2.60,7.62)
Do most shopping within walking distance from home (yes vs no)	1.19(0.88,1.60)	1.18(0.87,1.60)	1.83(1.13,2.95)	1.72(1.06,2.78)
Regularly walk a dog (yes vs no)	4.12(2.88,5.88)	4.41(3.05,6.37)	7.17(4.13,12.46)	7.76(4.39,13.73)

^1^Lubben scale, isolated <12.

^2^Geriatric Depression Scale, depressed >2.

^3^Self-rating of local area: Very Good/Good vs Average/poor.

^4^Self-rating of local area: safe vs unsafe.

^5^Model 1 = age + region + season + average monitor wear time, plus each variable in column, one at a time.

^6^Model 2 = age + region + season + average monitor wear time + depression + number of chronic conditions.

### Prevalence and correlates of attaining PA recommendations in bouts of 5 minutes or more

28% (n = 452) men and 21% (n = 177) achieved 150 minutes of MVPA defined as >1040 cpm in bouts of 5 minutes or more; (Additional file 1: Table S1). Correlates of achieving MVPA5+ were mostly similar to achieving MVPA10+, but also included social isolation, feeling safe in the daytime and doing shopping within walking distance of home. These associations were mostly confirmed in logistic regression models; (Additional file 1: Table S2, Model 1 and with additional adjustment for depression and number of chronic conditions (Model 2), stronger associations were seen for regularly walking a dog, leaving the house on more than 5 days per week, having no mobility limitations, not being depressed and, particularly for women, using active transport (walking or cycling).

### Attainment of PA recommendations using 1952 cpm cut-point

In further analyses using >1952 cpm to define MVPA, the prevalence of adherence to MVPA guidelines MVPA5+ was for 12% men and 8% for women, and MVPA10+ was 7% for men and 3% for women (Table 1). Correlates of adherence to MVPA10+ guidelines in men and women are presented in Additional file 1: Table S3. In logistic regression models, similar variables predicted adherence to MVPA10+ guidelines in men for 1952 cpm cut-point as for 1040 cpm cut-point and effects were in the same direction (Additional file 1: Table S4). The physical health (chronic conditions, mobility limitations), depression and the behavioural variables were all consistent correlates of achieving MVPA. Logistic regression models were not presented for women due to low numbers, only 3% (n = 26), attaining guidelines with the higher cut-point for MVPA (Additional file 1: Table S3).

## Discussion

This study, including a larger sample than previous studies [2,15] of objectively measured PA in older men and women, found that, using an older adult–specific cut-point of 1040 cpm to define MVPA, more than one in seven men and one in ten women aged 70–93 years accumulated the guideline amount (equivalent of 150 minutes of moderate to vigorous-intensity PA per week) in bouts lasting 10 minutes or more. However almost twice as many (28% men and 21% women) accumulated guideline amounts but in bouts lasting 5 minutes or more. Only 7% men and 3% women attained guideline amounts of PA in bouts lasting at least 10 minutes when the cut-point to define MVPA was raised to 1952 cpm. Our study includes more recent data and detailed information on a wider range of correlates than previous studies. Consistent correlates of achieving 150 minutes of MVPA in bouts of 10 minutes or more were not being depressed, or having mobility limitations outdoors and fewer chronic health conditions, having high exercise self-efficacy (confidence in the ability to be physically active) and believing that exercise is beneficial to health, leaving the house most days, regularly walking a dog, using active transport (walking or cycling), and feeling safe walking after dark in the local neighbourhood.

### Levels of PA in older people in the UK

Adherence to the PA guidelines was low using either intensity cut-point to define MVPA. Our estimates of 15% men and 10% women attaining guidelines based on the age appropriate cut-point of activity >1040 cpm to define MVPA were higher than previous studies have reported. However our estimates of 7% and 3% using the cut-point of >1952 cpm are more in line with estimates from other UK-based studies of older adults which have used cut-points nearer to 2000 cpm [2,15,17]. In the Health Survey for England subsample (approximately 400 adults with valid accelerometer data aged over 65), 5% men and 0% of women adhered to the guidelines [2]. In the OPAL study, among 230 community-dwelling adults in UK aged over 70 years, only 1.3% met the MVPA10+ (>1952 cpm) guidelines [15]. Another study (n = 240) of older adults recruited from UK primary care centres reported that 2.5% of men and women achieved 150 minutes or more per week of MVPA10+ (defined as >1999 cpm), while 62% did not do any minutes of MVPA in a week [17]. Data from National Health And Nutrition Examination Survey in the USA estimated that 6.3% of over 70 year olds met guidelines, counting activity in bouts of ≥10 minutes and 10.4% not considering bouts [27]. Whilst half of 70–74 year olds in the OPAL study accumulated 150 minutes/week of MVPA in bouts of one minute or more, this prevalence declined steeply with age to 7% of over 85 year olds [15]. These studies used the same brand of accelerometer to measure activity as we did, although there were differences in the data processing protocols and the cut-points to define MVPA (1952 cpm [15], 1999 cpm [17] and 2020 cpm [2]) whereas we used 1040 cpm or 1952 cpm.

### Importance of MVPA bout length

When 5 minute bouts were considered instead of 10 minute bouts, the prevalence of attaining 150 minutes/week of MVPA was doubled. The 10 minute bouts stipulated by guidelines were based on trial data for cardiometabolic risk factors only, not clinical end points [12]. Newer data indicate that accumulating 10 minute bouts of PA is associated with reduced risks of adiposity and intermediary markers of disease in older adults [28]. However, we lack data showing whether health benefits for hard clinical endpoints accrue from bouts of activity shorter than 10 minutes, or whether it is the total volume of PA which is important, irrespective of the pattern in which is accumulated. Until evidence from studies linking accelerometer data to incident morbidity and mortality data are available, it remains unclear whether older adults would benefit from higher cumulated total MVPA, or from increasing the duration of shorter spells of MVPA. Data from studies of high intensity training suggest that short high intensity interval training can have beneficial effects on CVD risk factors [29] but we do not know if such training regimes are sustainable over the long term. It is unlikely that high intensity training would be suitable for older adults, especially in unsupervised settings, particularly given the higher levels of co-morbidities, mobility limitations and balance impairments in that age-group.

### Correlates of meeting MVPA guidelines

The directions of associations between the correlates and adherence to MVPA guidelines fitted with expectations from studies with self-reported PA and previous smaller studies using accelerometers. As expected [17,30,31], we found that poor physical health (chronic health conditions and mobility limitations) was an important barrier to achieving MVPA guidelines. We found that associations with poor physical health remained after adjusting for depression. Depression itself was an important barrier to achieving MVPA guidelines, after accounting for presence of chronic physical conditions. Other modifiable factors which could be potential levers for PA interventions in older adults were identified; as seen elsewhere higher levels of exercise self- efficacy [14,17] and exercise outcomes expectations were associated with greater adherence to guidelines. Men left the house on more days than women and also reported walking or cycling for transport more often than women, and each of these behaviours, as well as dog-walking (as seen elsewhere [17]) consistently predicted achieving guidelines, suggesting that encouraging older adults to leave the house each day and do walking based activities could improve adherence to guidelines. Most participants had positive perceptions of their neighbourhood, and these were not strongly related to achieving guidelines, with the exception of feeling safe walking alone after dark, but very few women felt safe walking after dark. Other studies report that associations between neighbourhood deprivation measures and PA are mediated by personal factors [16].

### Strengths and limitations

To our knowledge, this is the largest study to have estimated adherence to national guidelines using accelerometer-measured PA in older British men and women including the oldest old. Previous studies included fewer participants and fewer correlates. We study community dwelling adults, not special high risk clinical populations, hence results should be generalizable to this age group. We used the GT3x, a widely used accelerometer. Given the lack of consensus about which cut-points to use to define MVPA in older adults, we tested different, validated, cut-points [22,23] to define MVPA levels. Our data are cross-sectional, so we cannot assess causality among the correlates of MVPA adherence, longitudinal studies will be necessary. Loss to follow-up may be greater among the more ill and infirm who are likely to be less active, resulting in under-representation of the less active. However, our response rates to the postal accelerometer study (51% in men and 29% in women) are line with other studies of older adults: 21% [15], 43% [17] and in the Health Survey for England 37% women and 48% men over 75 years had 4 or more days with valid data [2].

## Conclusions

Few adults currently adhere to PA guidelines for older people; 7% men and 3% women when using a cut-point to define MVPA validated in middle aged adults, rising to 15% of men and 10% of women when using an age-appropriate cut-point to define MVPA. Adherence is higher still (28% of men and 21% of women) when guidelines are relaxed to include bouts of MVPA lasting only 5 minutes or more. The low levels attaining guidelines as currently defined to include bouts of MVPA lasting 10 minutes or more highlight the huge potential for health improvement if the declines in PA levels between middle and older age could be reduced. Older adults could benefit from the wide-ranging health benefits of MVPA, if MVPA levels could be increased and potentially if the current pattern of more frequent short bouts could be changed to include longer bouts of more than 10 minutes duration. Future longitudinal studies using objective measures of PA in older adults are necessary to assess whether short (e.g., 5 minutes) and longer bouts of PA have equivalent health benefits for morbidity and mortality from CVD and other clinical end points. This cross sectional study cannot evaluate which patterns of accelerometer-measured PA are causally associated with better health outcomes. We identified a range of correlates adhering to PA guidelines but further longitudinal studies using objective measures of PA in older adults are now needed to assess causality amongst these correlates of MVPA.

## Abbreviations

PA: Physical activity; MVPA: Moderate to vigorous physical activity; MVPA5+: Moderate to vigorous physical activity in bouts lasting 5 minutes or more; MVPA10+: Moderate to vigorous physical activity in bouts lasting 10 minutes or more; CVD: Cardiovascular disease; MET: Metabolic equivalent of task; CPM: Counts per minute.

## Competing interests

The authors declare that they have no competing interests.

## Authors’ contributions

BJ conceived and designed the study, analysed and interpreted data and drafted the manuscript. BJ is the guarantor of the study. CS analysed and interpreted data, revised and approved of the final version of the paper, IML interpreted data, revised and approved of the final version of the paper, MC analysed and interpreted data and approved of the final version of the paper, AA provided data and approved of the final version of the paper, CG collected and interpreted data, revised and approved of the final version of the paper, JPC interpreted data, revised and approved of the final version of the paper, SA collected data, interpreted data, revised and approved of the final version of the paper, LTL collected data, interpreted data, revised and approved of the final version of the paper, SGW conceived and designed the study, interpreted data and revised and approved of the final version of the paper, PHW conceived and designed the study, collected and interpreted data and revised and approved of the final version of the paper. All authors read and approved the final manuscript.

## Pre-publication history

The pre-publication history for this paper can be accessed here:

http://www.biomedcentral.com/1471-2458/14/382/prepub

## Supplementary Material

Additional file 1: Table S1 Characteristics of participants according to attainment of 150 minutes/week MVPA (>1040 cpm) in bouts lasting 5 minutes or more. **Table S2.** Associations (odds ratio, [95% CI]) between participant characteristics and attaining ≥150 minutes of MVPA(>1040 cpm)/week in bouts ≥5 minutes, (n = 2426). **Table S3.** Characteristics of participants according to attainment of 150 minutes/week MVPA (>1952 cpm) in bouts lasting 10 minutes or more. **Table S4.** Associations (odds ratio, [95% CI]) between participant characteristics and attaining ≥150 minutes of MVPA (>1952 cpm)/ week in bouts lasting 10 minutes or more. Click here for file

## References

[B1] Lee IM Shiroma EJ Lobelo F Puska P Blair SN Katzmarzk PT Effect of physical inactivity on major non-communicable diseases worldwide: an analysis of burden of disease and life expectancy Lancet 2012 380 219 229 10.1016/S0140-6736(12)61031-9 22818936 PMC3645500

[B2] Craig R Mindell J Hirani V Health Survey for England 2008. Physical Activity and Fitness. Summary of Key findings 2009 London: The Health and Social Care Information Centre

[B3] Baptista F Santos DA Silva AM Mota J Santos R Vale S Ferreira JP Raimundo AM Moreira H Sardinha LB Prevalence of the Portuguese population attaining sufficient physical activity Med Sci Sports Exerc 2012 44 466 473 doi:10.1249/MSS.0b013e318230e441 10.1249/MSS.0b013e318230e441 21844823

[B4] Colley RC Garriguet D Janssen I Craig CL Clarke J Tremblay MS Physical activity of Canadian adults: accelerometer results from the 2007 to 2009 Canadian Health Measures Survey Health Rep 2011 22 7 14 21510585

[B5] Troiano RP Berrigan D Dodd KW Masse LC Tilert T McDowell M Physical activity in the United States measured by accelerometer Med Sci Sports Exerc 2008 40 181 188 10.1249/mss.0b013e31815a51b3 18091006

[B6] Ashe MC Miller WC Eng JJ Noreau L Older adults, chronic disease and leisure-time physical activity Gerontology 2009 55 64 72 10.1159/000141518 18566534 PMC3167824

[B7] Knuth AG Bacchieri G Victora CG Hallal PC Changes in physical activity among Brazilian adults over a 5-year period J Epidemiol Community Health 2010 64 591 595 10.1136/jech.2009.088526 19706621

[B8] Chief Medical Officers of England, Scotland, Wales and Northern Ireland Start Active, Stay Active. A report on physical activity for health from the four home countries’ Chief Medical Officers 2011 London: Crown

[B9] Nelson ME Rejeski J Blair SN Duncan PW Judge JO King AC Macera CA Castaneda-Sceppa C Physical Activity and Public Health in Older Adults: Recommendation From the American College of Sports Medicine and the American Heart Association Circulation 2007 116 1094 1105 17671236 10.1161/CIRCULATIONAHA.107.185650

[B10] DiPietro L Physical Activity in Aging: Changes in Patterns and Their Relationship to Health and Function J Gerontol A: Biol Med Sci 2001 56 13 22 10.1093/gerona/56.suppl_2.13 11730234

[B11] Washburn RA Assessment of physical activity in older adults Res Q Exerc Sport 2000 71 S79 S88 10925829

[B12] Physical Activity Guidelines Advisory Committee Physical Activity Guidelines Advisory Committee Report 2008 Washington, DC: US, Department of Health and Human Services 10.1111/j.1753-4887.2008.00136.x 19178654

[B13] Evenson KR Buchner DM Morland KB Objective measurement of physical activity and sedentary behavior among US adults aged 60 years or older Prev Chronic Dis 2012 9 E26 22172193 PMC3277387

[B14] Booth ML Owen N Bauman A Clavisi O Leslie E Social-cognitive and perceived environment influences associated with physical activity in older Australians Prev Med 2000 31 15 22 10.1006/pmed.2000.0661 10896840

[B15] Davis MG Fox KR Hillsdon M Sharp DJ Coulson JC Thompson JL Objectively measured physical activity in a diverse sample of older urban UK adults Med Sci Sports Exerc 2011 43 647 654 20689449 10.1249/MSS.0b013e3181f36196

[B16] Fox KR Hillsdon M Sharp D Cooper AR Coulson JC Davis M Harris R McKenna J Narici M Stathi A Thompson JL Neighbourhood deprivation and physical activity in UK older adults Health Place 2011 17 633 640 10.1016/j.healthplace.2011.01.002 21292536

[B17] Harris TJ Owen CG Victor CR Adams R Cook DG What factors are associated with physical activity in older people, assessed objectively by accelerometry? Br J Sports Med 2009 43 442 450 10.1136/bjsm.2008.048033 18487253

[B18] Walker M Whincup PH Shaper AG The British Regional Heart Study 1975–2004 Int J Epidemiol 2004 33 1185 1192 10.1093/ije/dyh295 15319395

[B19] Lawlor DA Bedford C Taylor M Ebrahim S Geographical variation in cardiovascular disease, risk factors, and their control in older women: British Women’s Heart and Health Study J Epidemiol Community Health 2003 57 134 140 10.1136/jech.57.2.134 12540690 PMC1732392

[B20] Choi L Liu Z Matthews C Buchowski MS Physical Activity: Process Physical Activity Accelerometer Data 2011 Nashville, TN: Leena Choi

[B21] Hart TL Swartz AM Cashin SE Strath SJ How many days of monitoring predict physical activity and sedentary behaviour in older adults? Int J Behav Nutr Phys Act 2011 8 62 10.1186/1479-5868-8-62 21679426 PMC3130631

[B22] Copeland JL Esliger DW Accelerometer assessment of physical activity in active, healthy older adults J Aging Phys Act 2009 17 17 30 19299836 10.1123/japa.17.1.17

[B23] Freedson PS Melanson E Sirard J Calibration of the Computer Science and Applications, Inc. accelerometer Med Sci Sports Exerc 1998 30 777 781 10.1097/00005768-199805000-00021 9588623

[B24] Van Marwijk HW Wallace P De Bock GH Hermans J Kaptein AA Mulder JD Evaluation of the feasibility, reliability and diagnostic value of shortened versions of the geriatric depression scale Br J Gen Pract 1995 45 195 199 7612321 PMC1239201

[B25] Lubben J Blozik E Gillmann G Iliffe S Von Renteln KW Beck JC Stuck AE Performance of an abbreviated version of the Lubben Social Network Scale among three European community-dwelling older adult populations Gerontologist 2006 46 503 513 10.1093/geront/46.4.503 16921004

[B26] Resnick B Jenkins LS Testing the Reliability and Validity of the Self-Efficacy for Exercise Scale Nurs Res 2000 49 154 159 10.1097/00006199-200005000-00007 10882320

[B27] Tucker JM Welk GJ Beyler NK Physical activity in U.S.: adults compliance with the Physical Activity Guidelines for Americans Am J Prev Med 2011 40 454 461 10.1016/j.amepre.2010.12.016 21406280

[B28] Gennuso KP Gangnon RE Matthews CE Thraen-Borowski KM Colbert LH Sedentary Behavior, Physical Activity, and Markers of Health in Older Adults Med Sci Sports Exerc 2013 45 8 1493 1500 doi:10.1249/MSS.0b013e318288a1e5. 10.1249/MSS.0b013e318288a1e5 23475142 PMC5764165

[B29] Gibala MJ Little JP MacDonald MJ Hawley JA Physiological adaptations to low-volume, high-intensity interval training in health and disease J Physiol 2012 590 1077 1084 10.1113/jphysiol.2011.224725 22289907 PMC3381816

[B30] Chipperfield JG Newall NE Chuchmach LP Swift AU Haynes TL Differential Determinants of Men’s and Women’s Everyday Physical Activity in Later Life J Gerontol B Psychol Sci Soc Sci 2008 63 S211 S218 10.1093/geronb/63.4.S211 18689770 PMC3874240

[B31] Booth ML Bauman A Owen N Gore CJ Physical activity preferences, preferred sources of assistance, and perceived barriers to increased activity among physically inactive Australians Prev Med 1997 26 131 137 10.1006/pmed.1996.9982 9010908

