# Supplementary material for: Adherence to physical activity guidelines in older adults, using objectively measured physical activity in a population-based study
Source: BMC Public Health. 2014 Apr 19;14:382. doi: 10.1186/1471-2458-14-382 (PMC4021412; doi:10.1186/1471-2458-14-382)
Supplement: Additional file 1: Table S1 — Characteristics of participants according to attainment of 150 minutes/week MVPA (>1040 cpm) in bouts lasting 5 minutes or more. Table S2. Associations (odds ratio, [95% CI]) between participant characteristics and attaining ≥150 minutes of MVPA(>1040 cpm)/week in bouts ≥5 minutes, (n = 2426). Table S3. Characteristics of participants according to attainment of 150 minutes/week MVPA (>1952 cpm) in bouts lasting 10 minutes or more. Table S4. Associations (odds ratio, [95% CI]) between participant characteristics and attaining ≥150 minutes of MVPA (>1952 cpm)/ week in bouts lasting 10 minutes or more. [file 1471-2458-14-382-S1.docx]

**Supplementary Table 1.** **Characteristics of participants according to attainment of 150 minutes/week MVPA (>1040cpm) in bouts lasting 5 minutes or more.**

|  | **Men** | |  | **Women** | |  |
| --- | --- | --- | --- | --- | --- | --- |
|  | **<150 mins/ week MVPA** | **≥150 mins/ week MVPA** | **p-value** | **<150 mins/ week MVPA** | **≥150 mins/ week MVPA** | **p-value** |
| N, % (n) | 71.6(1141) | 28.4(452) |  | 79.3(680) | 20.6(177) |  |
| Age (years), %(n) |  |  | <0.001 |  |  | <0.001 |
| 70-75 | 24.2(276) | 38.5(174) |  | 32.9(224) | 59.3(105) |  |
| 75-80 | 37.7(430) | 41.8(189) |  | 30.4(207) | 28.8(51) |  |
| 80-85 | 26.2(299) | 15.3(69) |  | 25.3(172) | 9(16) |  |
| Over 85 | 11.9(136) | 4.4(20) |  | 11.3(77) | 2.8(5) |  |
| Living alone vs living with others, %(n) | 22.1(249) | 19.9(89) | 0.350 | 41.4(278) | 41.7(73) | 0.934 |
| **Physical Health** |  |  |  |  |  |  |
| ≥ 3 chronic conditions, %(n) | 15.1(172) | 5.3(24) | <0.001 | 11.8(80) | 6.8(12) | <0.001 |
| Falls in the past 12 months, %(n) |  |  | <0.001 |  |  | 0.066 |
| One fall | 8.6(97) | 10.4(46) |  | 11.7(78) | 7.4(13) |  |
| ≥2 Falls | 14.4(162) | 5.2(23) |  | 10.5(70) | 6.8(12) |  |
| Moderate/severe mobility limitations outdoors, %(n) | 18.5(207) | 2.9(13) | <0.001 | 17.9(120) | 1.1(2) | <0.001 |
| **Mental health and wellbeing** |  |  |  |  |  |  |
| Exercise Self Efficacy, (z-scores), (mean, SD) | -0.20(1.00) | 0.52(0.77) | <0.001 | -0.14(0.97) | 0.63(0.80) | <0.001 |
| Exercise Outcome Expectations, (z-scores), (mean, SD) | -0.15(1.01) | 0.36(0.87) | <0.001 | -0.08(0.97) | 0.47(0.77) | <0.001 |
| Socially isolated, %(n) | 24.6(278) | 17.8(80) | 0.004 | 22(149) | 14.2(25) | 0.022 |
| Depressed (Geriatric Depression Scale), %(n) | 28.3(320) | 9.3(42) | <0.001 | 24.3(164) | 7.3(13) | <0.001 |
| **Environment** |  |  |  |  |  |  |
| Social and leisure activities, %(n)^1^ | 46.9(516) | 59.7(265) | <0.001 | 54.8(364) | 68.9(122) | <0.001 |
| Facilities for people your age, %(n)^1^ | 40(434) | 50.3(224) | <0.001 | 59.6(399) | 75(132) | <0.001 |
| Local transport to where you want to go, %(n)^1^ | 67.8(743) | 73.1(323) | 0.042 | 72.8(488) | 77.7(136) | 0.191 |
| The area has a nice place to go for a walk, %(n)^1^ | 80.4(901) | 88.9(400) | <0.001 | 77.2(516) | 89.3(158) | <0.001 |
| Feel safe walking alone in the daytime, %(n)^1^ | 95.7(1075) | 98.2(442) | 0.016 | 95(640) | 98.3(174) | 0.052 |
| Feel safe walking alone after dark, %(n)^1^ | 65(727) | 83.7(374) | <0.001 | 30.7(207) | 55.4(98) | <0.001 |
| **Behaviours** |  |  |  |  |  |  |
| Leave the house ≥5 days /past week, %,(n) | 79.3(875) | 96.8(428) | <0.001 | 66.9(444) | 97.1(165) | <0.001 |
| Cycle/walk regularly, %(n) | 48.6(554) | 72.3(327) | <0.001 | 7.1(48) | 32.8(58) | <0.001 |
| Do most shopping walking distance from home, %(n) | 31.3(346) | 38.1(169) | 0.005 | 30.7(207) | 45.1(79) | <0.001 |
| Regularly walks a dog, %(n) | 6.8(77) | 22.4(100) | <0.001 | 5.3(36) | 25.4(45) | <0.001 |

1 Self-rating of local area as “good” or “very good”

**Supplementary Table 2**. **Associations (odds ratio,[95%CI]) between participant characteristics and attaining ≥150 minutes of MVPA(>1040cpm)/week in bouts ≥5 minutes, (n=2426).**

|  | **Men** | | **Women** | |
| --- | --- | --- | --- | --- |
|  | **Model 1 ^5^** | **Model 2 ^6^** | **Model 1 ^5^** | **Model 2 ^6^** |
| **Physical Health** |  |  |  |  |
| No Chronic conditions vs ≥1 condition (baseline) | 1.99(1.58,2.52) | 1.89(1.49,2.39) | 1.96(1.36,2.82) | 1.82(1.26,2.64) |
| One fall vs no falls in past 12 months | 1.29(0.87,1.91) | 1.39(0.93,2.07) | 0.61(0.32,1.18) | 0.68(0.35,1.32) |
| ≥2 falls vs no falls in past 12 months | 0.40(0.25,0.63) | 0.52(0.32,0.84) | 0.72(0.37,1.43) | 0.97(0.48,1.94) |
| No/ slight mobility limitations vs moderate or more | 6.27(3.51,11.19) | 3.99(2.19,7.27) | 15.15(3.53,65.02) | 9.74(2.28,41.61) |
| **Mental health and wellbeing** |  |  |  |  |
| Exercise Self Efficacy, (z-scores, 1-SD increase) | 2.21(1.92,2.55) | 2.03(1.75,2.35) | 2.11(1.71,2.60) | 1.95(1.57,2.43) |
| Exercise Outcome Expectations, (z-scores, 1-SD increase) | 1.69(1.49,1.93) | 1.57(1.38,1.80) | 1.80(1.43,2.25) | 1.67(1.32,2.12) |
| Not socially isolated vs isolated^1^ | 1.31(0.98,1.75) | 1.20(0.89,1.62) | 1.13(0.69,1.85) | 0.84(0.50,1.41) |
| Not depressed vs depressed ^2^ | 3.10(2.18,4.40) | 2.92(2.05,4.17) | 3.35(1.81,6.22) | 3.11(1.67,5.78) |
| **Environment** |  |  |  |  |
| Social and leisure activities^3^ | 1.52(1.20,1.92) | 1.34(1.06,1.71) | 1.67(1.15,2.43) | 1.53(1.04,2.24) |
| Facilities for people your age^3^ | 1.38(1.09,1.74) | 1.22(0.96,1.55) | 1.88(1.26,2.79) | 1.69(1.13,2.54) |
| Local transport^3^ | 1.29(0.99,1.66) | 1.22(0.94,1.59) | 1.24(0.81,1.89) | 1.13(0.73,1.74) |
| The area has somewhere nice to go for a walk^3^ | 1.65(1.17,2.31) | 1.41(0.99,2.00) | 2.08(1.22,3.55) | 1.94(1.12,3.35) |
| Feel safe when walking alone in the daytime^4^ | 2.03(0.93,4.42) | 1.48(0.66,3.32) | 1.90(0.55,6.56) | 1.47(0.41,5.24) |
| Feel safe when walking alone after dark^4^ | 2.40(1.80,3.20) | 2.14(1.59,2.87) | 2.32(1.62,3.33) | 2.07(1.43,2.99) |
| **Behaviours** |  |  |  |  |
| Leave house ≥5 days/week vs < 5 days/week | 6.25(3.56,10.96) | 5.06(2.86,8.97) | 11.51(4.58,28.95) | 9.29(3.68,23.44) |
| Cycle/walk vs use Car/Public transport | 2.50(1.94,3.21) | 2.14(1.66,2.77) | 7.12(4.41,11.51) | 6.36(3.92,10.31) |
| Do most shopping within walking distance from home (yes vs no) | 1.37(1.08,1.76) | 1.39(1.08,1.78) | 1.95(1.35,2.82) | 1.82(1.25,2.64) |
| Regularly walk a dog (yes vs no) | 3.68(2.63,5.16) | 3.97(2.80,5.63) | 4.73(2.86,7.83) | 5.09(3.01,8.59) |

^1^ Lubben scale, isolated <12

^2^ Geriatric Depression Scale, depressed >2

^3^ Self-rating of local area: Very Good/Good vs Average/poor

^4^ Self-rating of local area: safe vs unsafe

^5^ Model 1 = age + region + season + average monitor wear time, plus each variable in column, one at a time

^6^ Model 2 = age + region + season + average monitor wear time + depression + number of chronic conditions

**Supplementary Table 3 Characteristics of participants according to attainment of 150 minutes/week MVPA (>1952 cpm) in bouts lasting 10 minutes or more.**

|  | Men, MVPA<150 | Men, MVPA>=150 | p-value | Women, MVPA<150 | Women, MVPA>=150 | p-value |
| --- | --- | --- | --- | --- | --- | --- |
| N, % (n) | 92.7(1476) | 7.3(117) |  | 97.0(831) | 3.0(26) |  |
| Age (years), %(n) |  |  | 0.001 |  |  | 0.852 |
| 70-75 | 27.2(402) | 41(48) |  | 38.1(317) | 46.2(12) |  |
| >75-80 | 38.7(571) | 41(48) |  | 30.1(250) | 30.8(8) |  |
| >80-85 | 23.8(351) | 14.5(17) |  | 22.1(184) | 15.4(4) |  |
| Over 85 | 10.3(152) | 3.4(4) |  | 9.6(80) | 7.7(2) |  |
| Living alone vs living with others, %(n) | 21.2(310) | 24.3(28) | 0.431 | 40.9(336) | 57.7(15) | 0.088 |
| **Physical Health** |  |  |  |  |  |  |
| ≥ 3 chronic conditions, %(n) | 12.9(191) | 4.3(5) | <0.001 | 11.1(92) | 0(0) | 0.022 |
| Falls in the past 12 months, %(n) |  |  | 0.024 |  |  | 1.000 |
| One fall | 8.9(129) | 12.1(14) |  | 10.8(88) | 11.5(3) |  |
| ≥2 Falls | 12.4(180) | 4.3(5) |  | 9.8(80) | 7.7(2) |  |
| Moderate/severe mobility limitations outdoors, %(n) | 15.1(218) | 1.7(2) | <0.001 | 14.9(122) | 0(0) | 0.038 |
| **Mental health and wellbeing** |  |  |  |  |  |  |
| Exercise Self Efficacy, (z-scores), (mean, SD) | -0.05(1.00) | 0.73(0.60) | <0.001 | 0.00(0.98) | 1.00(0.74) | <0.001 |
| Exercise Outcome Expectations, (z-scores), (mean, SD) | -0.06(1.00) | 0.57(0.88) | <0.001 | 0.02(0.95) | 0.73(0.64) | <0.001 |
| Socially isolated, %(n) | 22.5(330) | 23.9(28) | 0.729 | 20.2(167) | 28(7) | 0.321 |
| Depressed (Geriatric Depression Scale), %(n) | 24.3(356) | 5.2(6) | <0.001 | 20.9(173) | 15.4(4) | 0.628 |
| **Environment** |  |  |  |  |  |  |
| Social and leisure activities, %(n)^1^ | 50.2(717) | 55.7(64) | 0.258 | 57.5(469) | 65.4(17) | 0.426 |
| Facilities for people your age, %(n)^1^ | 42.9(606) | 44.8(52) | 0.680 | 62.3(511) | 76.9(20) | 0.129 |
| Local transport to where you want to go, %(n)^1^ | 69.3(986) | 69(80) | 0.933 | 73.7(604) | 80(20) | 0.645 |
| The area has a nice place to go for a walk, %(n)^1^ | 82.1(1194) | 91.5(107) | 0.010 | 79.5(651) | 88.5(23) | 0.329 |
| Feel safe walking alone in the daytime, %(n)^1^ | 96.4(1403) | 97.4(114) | 0.794 | 95.5(788) | 100(26) | 0.623 |
| Feel safe walking alone after dark, %(n)^1^ | 69(1000) | 86.3(101) | <0.001 | 95.5(788) | 61.5(16) | 0.006 |
| **Behaviours** |  |  |  |  |  |  |
| Leave the house ≥5 days /past week, %,(n) | 83.4(1192) | 95.7(111) | <0.001 | 72.4(586) | 92(23) | 0.037 |
| Cycle/walk regularly, %(n) | 53.2(785) | 82.1(96) | <0.001 | 11.3(94) | 46.2(12) | <0.001 |
| Do most shopping walking distance from home, %(n) | 32.6(467) | 41.4(48) | 0.060 | 33.4(275) | 42.3(11) | 0.602 |
| Regularly walks a dog, %(n) | 10.3(150) | 23.9(27) | <0.001 | 8.7(72) | 34.6(9) | <0.001 |

^1^ Self-rating of local area as “good” or “very good”

**Supplementary Table 4. Associations (odds ratio,[95%CI]) between participant characteristics and attaining ≥150 minutes of MVPA (>1952cpm)/ week in bouts lasting 10 minutes or more.**

|  | **Men** | |
| --- | --- | --- |
|  | **Model 1 ^5^** | **Model 2 ^6^** |
| **Physical Health** |  |  |
| No Chronic conditions vs ≥1 condition (baseline) | 1.92(1.30,2.83) | 1.80(1.22,2.65) |
| One fall vs no falls in past 12 months | 1.55(0.85,2.84) | 1.71(0.93,3.15) |
| ≥2 falls vs no falls in past 12 months | 0.40(0.16,1.01) | 0.56(0.22,1.42) |
| No/ slight mobility limitations vs moderate or more | 8.43(2.06,34.52) | 4.66(1.11,19.58) |
| **Mental health and wellbeing** |  |  |
| Exercise Self Efficacy, (z-scores, 1-SD increase) | 2.62(2.00,3.44) | 2.39(1.81,3.16) |
| Exercise Outcome Expectations, (z-scores, 1-SD increase) | 1.86(1.50,2.29) | 1.72(1.38,2.13) |
| Not socially isolated vs isolated^1^ | 0.80(0.51,1.25) | 0.70(0.44,1.11) |
| Not depressed vs depressed ^2^ | 4.90(2.12,11.32) | 4.61(1.99,10.65) |
| **Environment** |  |  |
| Social and leisure activities^3^ | 1.13(0.76,1.66) | 0.97(0.65,1.44) |
| Facilities for people your age^3^ | 0.98(0.66,1.44) | 0.84(0.57,1.25) |
| Local transport^3^ | 0.97(0.64,1.47) | 0.91(0.60,1.40) |
| The area has somewhere nice to go for a walk^3^ | 2.02(1.04,3.95) | 1.72(0.87,3.38) |
| Feel safe when walking alone in the daytime^4^ | 1.26(0.38,4.15) | 0.88(0.26,3.01) |
| Feel safe when walking alone after dark^4^ | 2.53(1.47,4.38) | 2.24(1.29,3.88) |
| **Behaviours** |  |  |
| Leave house ≥5 days/week vs < 5 days/week | 3.54(1.42,8.84) | 2.58(1.02,6.51) |
| Cycle/walk vs use Car/Public transport | 3.45(2.11,5.64) | 2.89(1.76,4.75) |
| Do most shopping within walking distance from home (yes vs no) | 1.41(0.95,2.11) | 1.40(0.94,2.10) |
| Regularly walk a dog (yes vs no) | 2.41(1.49,3.88) | 2.47(1.52,4.00) |

^1^ Lubben scale, isolated <12

^2^ Geriatric Depression Scale, depressed >2

^3^ Self-rating of local area: Very Good/Good vs Average/poor

^4^ Self-rating of local area: safe vs unsafe

^5^ Model 1 = age + region + season + average monitor wear time, plus each variable in column, one at a time

^6^ Model 2 = age + region + season + average monitor wear time + depression + number of chronic conditions
